# Calculating and Reporting Coefficients of Variation for DIA-Based Proteomics

**DOI:** 10.1021/acs.jproteome.4c00461

**Published:** 2024-11-22

**Authors:** Alejandro J. Brenes

**Affiliations:** † Centre for Inflammation Research, Institute for Regeneration and Repair, University of Edinburgh, Edinburgh EH16 4UU, United Kingdom

**Keywords:** proteomics, coefficient of variation, CV, precision, quantification

## Abstract

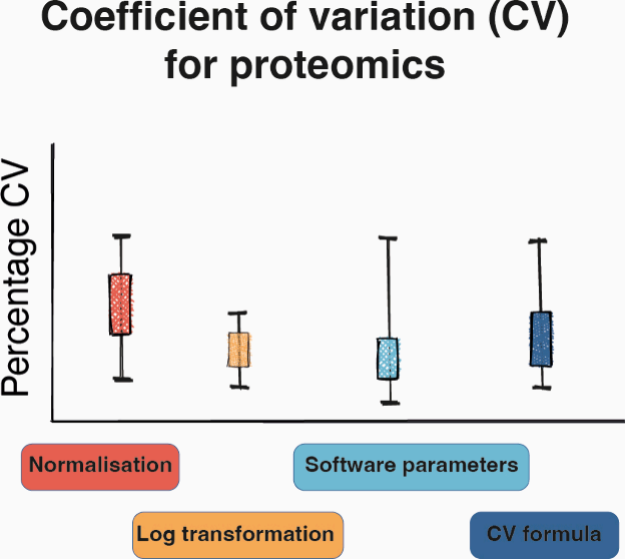

The coefficient of variation (CV) is a measure that is frequently used to assess data dispersion for mass spectrometry-based proteomics. In the current era of burgeoning technical developments, there has been an increased focus on using CVs to measure the quantitative precision of new methods. Thus, it has also become important to define a set of guidelines on how to calculate and report the CVs. This perspective shows the effects that the CV equation, data normalization as well as software parameters, can have on data dispersion and CVs, highlighting the importance of reporting all these variables within the methods section. It also proposes a set of recommendations to calculate and report CVs for technical studies, where the main objective is to benchmark technical developments with a focus on precision. To assist in this process, a novel R package to calculate CVs (proteomicsCV) is also included.

## Introduction

Proteomics aims to characterize all proteins present in a specific cell, cell type, tissue, or organism.^1^ Recent breakthroughs have enabled a robust coverage of proteomes at scale, detecting over 7,000 proteins in less than 30 min.^2,3^ Hence, the field is currently at an exciting stage where there is a drive to produce novel workflows and technological developments catered toward the high throughput analysis of low sample amounts, ranging down to individual single cells. This has also produced a renewed interest in evaluating the quantitative precision of such workflows, instruments, or software packages, where a common practice is to measure and highlight the coefficient of variation (CV).

The CV is a measure of dispersion and a relative measure of variability. It compares the standard deviation to the measured mean. For proteomics in essence, it is comparing how close multiple measurements from different samples are to each other. It is regularly used to assess the reproducibility of quantitative measurements, as the smaller the CV, the lower the deviation compared to the mean and the lower the dispersion; thus, lower CVs are equated to increased reproducibility and increased data quality. However, a lower CV is not always positive, as it has been reported that methodological issues such as faulty MS1 signal extraction or loose FDR can provide a lower but meaningless CVs.^4^ As such, it is important to consider these pitfalls when using the CVs to evaluate the quantification.

Current developments focused on technological advancements, including novel mass spectrometers,^2,5^ new sample handling and processing workflows,^5−11^ or updated software tools,^12,13^ frequently leverage CVs to evaluate the quantitative performance of their method. As such, it would be beneficial to set up some guidelines on how to calculate and report the CVs for such studies. Using the Kawashima^14^ data set, this perspective exemplifies how different the different CV formulas, data normalization steps as well as software parameters affect the CVs produced. The reanalyses presented here are openly available at PRIDE^15^ under accession PXD052403. Using this same data set, a set of guidelines are suggested to calculate CVs in studies focusing on technical/technological developments and a different set of recommendations are made for biological studies. Furthermore, to assist in calculating the CVs in a standardized manner an R package (proteomicsCV) is provided to assist in the process.

## The Effect of Data Normalization and Software Parameters on CVs

**Figure 1:**
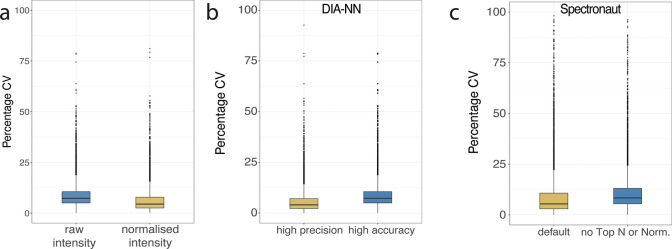
Normalization effect on the CV and data distributions: (a) Boxplots comparing the coefficient of variation (CV) produced by using raw intensity compared to median normalized intensity. (b) Boxplots showing the CVs produced by DIA-NN when using the High precision vs High accuracy Quantification strategy. (c) Boxplots showing the CVs produced by Spectronaut when using the default versus stringent settings. The top whisker extends from the hinge to the largest value no further than 1.5 × IQR from the hinge; the bottom whisker extends from the hinge to the smallest value at most 1.5 × IQR of the hinge.

Data normalization is an important step in the proteomic data analysis pipeline, which aims to remove systematic bias. As such, it also has a significant effect on the CVs that are produced and multiple tools have been developed to study this.^16^ To visualize the effect of data normalization, the Kawashima data was analyzed in DIA-NN^17^ 1.8 setting the “Quantification strategy” to high accuracy. The data was then analyzed with and without a median normalization strategy. As expected, the normalized data produces a median CV that is considerably lower than if CVs were calculated on the raw non-normalized intensity data (Figure 1a). With this effect in mind, it is of clear importance to specify the normalization procedures applied before calculating the CVs, and currently this is frequently omitted from the methods section. Furthermore, many software tools apply an array of data transformations by default, in some cases without users understanding that this is the case, which makes comparison of CVs challenging.

The automatic normalization produced with DIA software is not tool specific and can occur both in DIA-NN^17^ and Spectronaut.^18^ When processing DIA data with DIA-NN 1.8, there are two quantification options, “High Precision” and “High Accuracy”. The “High Precision” option in the for quantification menu produces a median CV that is 45% lower than the “High Accuracy” option (Figure 1b). These results are due to a considerably less aggressive bias removal procedure that is used within the “High Precision” option. The results from the “High Precision” option are very similar to the “High Accuracy” mode with an additional median normalization step applied to the data (Figure 1a). Understanding the differences between these two quantification modes in DIA-NN is an important consideration to comprehend and interpret the CVs that are produced. It should be noted new options are now available in DIA-NN 1.9 and the most similar alternative to “High Accuracy” in DIA-NN 1.8 is the “legacy (direct)” option.

Similarly, the default parameters that are preset in Spectronaut also include steps that minimize data dispersion and have an important effect on the CVs. The most important parameters that affect dispersion include a filter for the top 3 most abundant precursors and peptides, an operation that produces a CV almost as low as iBAQ,^19^ as well as the default application of a global data normalization step when the data set is less than 500 raw files. This global normalization is equivalent to the median normalization step applied to the DIA-NN data (Figure 1a). Disabling the Top 3 and data normalization options from the default Spectronaut parameters produces a CV that is 35% higher that using the default parameters (Figure 1c), similar to the effect observed with DIA-NN with the two quantification modes. As with DIA-NN it is important to specify the parameter selection explicitly, specifying the Top N filter and the normalization strategy instead of just stating the default parameters were used, this will ensure readers are aware of the transformations that are applied to the data before the CVs are calculated.

Thus, with the two most popular DIA software processing tools it is important to understand how the choice of parameters will affect the underlying data and to report these specific parameters accordingly to help interpret the CVs that are produced. It is argued here that there are specific situations where using each of the parameter options can be beneficial and others where it is not.

## Matching the CV Formula to the Appropriate Data

Regardless of the type of study or normalization step, it is important to understand how to calculate the CVs. There are two main formulas (see below) that are frequently used to calculate CVs for intensity-based proteomic data. It is important to know when it is appropriate to use each specific formula before applying it to the data.
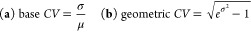


CV Formulas: (a) Base CV formula where σ is the standard deviation, and μ is the mean protein intensity across the samples. (b) Geometric CV formula where σ is the standard deviation of the log converted variance and *e* is Euler’s number (∼2.718).

**Figure 2:**
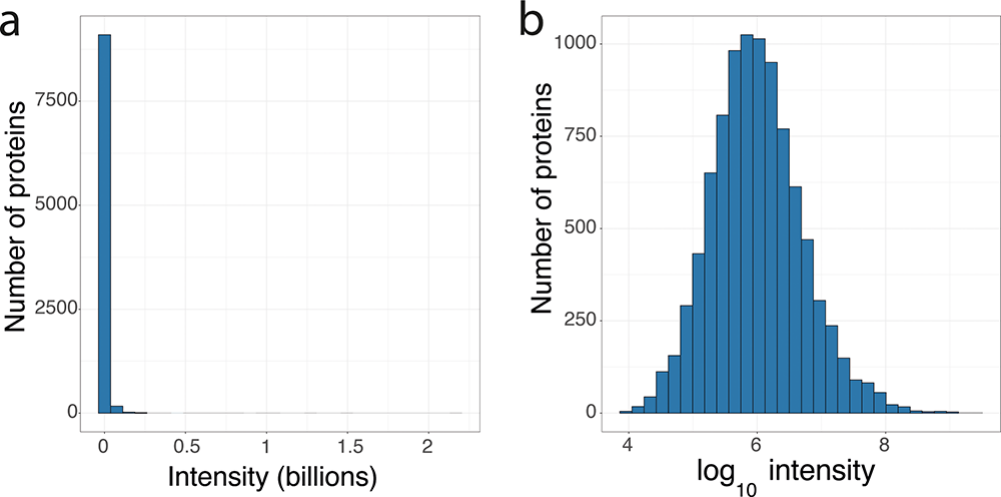
Log transforming intensity data: (a) Histogram showing the distribution of the raw intensity data. (b) Histogram showing the distribution of the log_10_ transformed intensity data.

**Figure 3:**
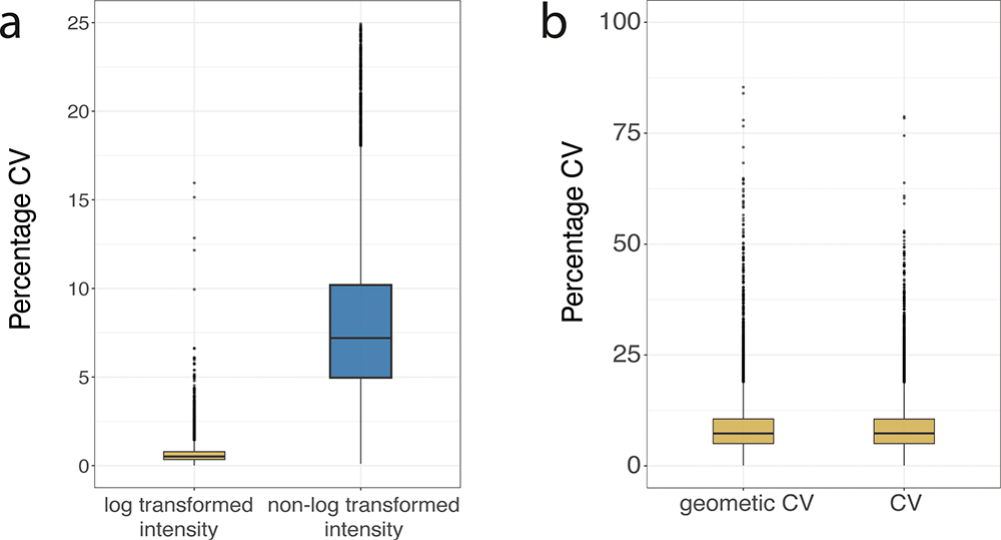
CVs across formulas: (a) Boxplots comparing the coefficient of variation (CV) produced by using the base formula with log transformed intensity and raw intensity. Y axis is cut at 25% to aid data visualization. (b) Boxplot showing the CVs produced by using log transformed data calculated with the geometric CV formula and raw intensity data calculated with the base CV formula (CV). For all boxplots, the bottom and top hinges represent the first and third quartiles. The top whisker extends from the hinge to the largest value no further than 1.5 × IQR from the hinge; the bottom whisker extends from the hinge to the smallest value at most 1.5 × IQR of the hinge.

Mass spectrometry-based intensity data are not normally distributed (Figure 2a). The intensity becomes approximately normally distributed only after performing a logarithmic transformation (Figure 2b). Hence, a common error is to apply a logarithmic conversion to the intensity data, while using the base CV formula on log transformed data. This erroneous use of the CV formula produces dramatically different results than when applying it to the nonlog transformed intensity. The resulting median CV can be >14 times lower than that of the raw intensity with 75% of all proteins detected with a CV < 0.75% (Figure 3a). This result is an artificial compression of the dispersion and does not faithfully represent the data acquired.

To avoid this artificial shrinkage, using the base formula with log transformed data should be avoided. Hence, for proteomic experiments the base CV formula should be applied only to nonlog transformed intensity. The geometric CV formula can also be utilized, but exclusively on log transformed data. When both the previously formulas are correctly applied to the right type of data, the median CV produced are virtually identical and are more reflective of the underlying data (Figure 3b).

## ProteomicsCV: An R Package To Calculate CVs

In an effort to simplify the CV calculation process for mass spectrometry-based proteomics data, an R package called “proteomicsCV” has been created and made openly available via the Comprehensive R Archive Network (https://cran.r-project.org/web/packages/proteomicsCV/). The package has a function to calculate the CVs for log transformed data, ‘protLogCV()’, and one function to calculate the CVs on nonlog transformed data ‘protCV()’. The package can be easily installed by running the following command in R: “install.packages(“proteomicsCV”)”.

## Summary and Recommendations

This perspective focuses on the use of CVs for proteomics. First, it is important to highlight that CVs themselves are a controversial measure, as they provide no insights into accuracy or the linearity of the quantitation. Furthermore, inappropriate parameters can also produce low CVs that do not reflect high data quality, as such they should be used with caution and overreliance on them is not recommended.^4^ Multiple options exist to assess the quantitative performance and quality control of proteomics experiments beyond simply relying on CVs.^20−22^

However, it is undeniable that CVs are frequently used in proteomics. Thus, for studies that make use of CVs this perspective provides some tools and recommendations. First, all studies should specify how the resulting CVs were calculated. Hence, the methods section should state the formula that was used to calculate the CV as well as any data normalization or data transformation steps that were performed before the CVs were produced. CVs should be calculated using the nonlog transformed intensities with the base formula or the log transformed intensities with the geometric CV formula.

The remaining recommendations differ for technical/technological studies and for biological experiments. For technical studies whose purpose is to benchmark the reproducibility in the quantification of a new instrument or method, and where the samples measured are mostly technical replicates it is recommended to use the non-normalized intensity data with no additional transformations or normalization to calculate the CVs. This it will provide the most realistic overview of the dispersion present in the data. Therefore, for these technical studies, specific parameters for DIA-NN and Spectronaut are recommended. When using DIA-NN < 1.9 it is suggested to select the “high accuracy” mode within the “Quantification strategy” dropdown menu. For DIA-NN 1.9 onward it is recommended to use the “legacy (direct)” option. When using Spectronaut it is recommended to alter the default parameters by disabling the Top N (with 3 as default) for both major and minor groupings, and to disable the automatic data normalization step. All the previously mentioned settings should be specified within the methods section of the relevant publications. The normalization and transformations can minimize the differences in instrument performance, such as negating an increase in total intensity detected, therefore the raw intensity can be the most informative input when analyzing performance in these technical studies.

For experiments aiming to explore the biology of different cells, cell types, or conditions, with different biological replicates and whose primary objective is not to test reproducibility of the data produced by the mass spectrometer, then it is sensible to calculate the CVs on the normalized data. Technical replicates in proteomics are expected to be more homogeneous as they tend to be multiple measurements of the same starting sample. Biological replicates on the other hand are expected to be much more heterogeneous, as they are measurements from different samples. Biological replicares are frequently derived from different individuals where it is known protein abundance can change considerably from sample to sample, especially when working with ex-vivo material. Hence, calculating the CVs on normalized data for biological studies will provide a better overview of the data set that will be used to analyze the biological dimension. As such it is reasonable to use “High Precision” within DIA-NN 1.8. Furthermore, it is highly recommended to use the new QuantUMS options in DIA-NN 1.9 instead of the “legacy (direct)” option meant for technical studies. Similarly, it is also perfectly reasonable to enable normalization within Spectronaut. However, the use of these parameters should be clearly stated within the methods, so readers are aware of the transformations applied before the CVs are calculated, and like with technical studies, the formula used to calculate the CVs should be clearly stated in the methods.

This perspective provides recommendations to calculate CVs as well as an R package (proteomicsCV) to assist in the process of correctly calculating these CVs. Specific parameters are recommended for biological studies, reflecting the heterogeneity of the samples in such studies. Similarly, specific recommendations are provided for technical studies with the aim of improving the cross-comparability of CVs as well as better reflecting the underlying data dispersion and instrument performance. Recent studies on similar instrumentation platforms have produced vastly different CVs, likely reflecting distinct data processing steps before they are calculated; however, most of the studies do not document the process sufficiently to determine if this is the case. This work aims to assist researchers in avoiding this scenario.

## Data Availability

The raw files, the FASTA file, the processed search results from DIA-NN using the two distinct quantification strategies, and the two distinct Spectronaut searches are available on ProteomeXchange^23^ via PRIDE^15^ under the identifier PXD052403 (https://www.ebi.ac.uk/pride/archive/projects/PXD052403). The R package proteomicsCV is freely available on CRAN https://cran.r-project.org/web/packages/proteomicsCV/index.html and can be installed in R using the ‘install.packages(“proteomicsCV”)’ command.
